# Influence of Multiple Donor Renal Arteries on the Outcome and Graft Survival in Deceased Donor Kidney Transplantation

**DOI:** 10.3390/jcm10194395

**Published:** 2021-09-26

**Authors:** Uwe Scheuermann, Sebastian Rademacher, Tristan Wagner, Andri Lederer, Hans-Michael Hau, Daniel Seehofer, Robert Sucher

**Affiliations:** 1 Department of Visceral, Transplantation, Vascular and Thoracic Surgery, University Hospital of Leipzig, 04103 Leipzig, Germany; sebastian.rademacher@medizin.uni-leipzig.de (S.R.); tristan.wagner@ukmuenster.de (T.W.); andri.lederer@medizin.uni-leipzig.de (A.L.); daniel.seehofer@medizin.uni-leipzig.de (D.S.); robert.sucher@medizin.uni-leipzig.de (R.S.); 2 Department of General, Visceral and Transplant Surgery, University Hospital Münster, 48149 Münster, Germany; 3 Department of Visceral, Thoracic and Vascular Surgery, University Hospital Carl-Gustav-Carus, TU Dresden, 01307 Dresden, Germany; hans-michael.hau@uniklinikum-dresden.de

**Keywords:** kidney transplant, multiple arteries, anastomosis, reconstruction, outcome, delayed graft function, warm ischemia time, cold ischemia time, survival

## Abstract

Aim: Complex arterial reconstruction in kidney transplantation (KT) using kidneys from deceased donors (DD) warrants additional study since little is known about the effects on the mid- and long-term outcome and graft survival. Methods: A total of 451 patients receiving deceased donor KT in our department between 1993 and 2017 were included in our study. Patients were divided into three groups according to the number of arteries and anastomosis: (A) 1 renal artery, 1 arterial anastomosis (*N* = 369); (B) >1 renal artery, 1 arterial anastomosis (*N* = 47); and (C) >1 renal artery, >1 arterial anastomosis (*N* = 35). Furthermore, the influence of localization of the arterial anastomosis (common iliac artery (CIA), versus non-CIA) was analyzed. Clinicopathological characteristics, outcome, and graft and patient survival of all groups were compared retrospectively. Results: With growing vascular complexity, the time of warm ischemia increased significantly (groups A, B, and C: 40 ± 19 min, 45 ± 19 min, and 50 ± 17 min, respectively; *p* = 0.006). Furthermore, the duration of operation was prolonged, although this did not reach significance (groups A, B, and C: 175 ± 98 min, 180 ± 35 min, and 210 ± 43 min, respectively; *p* = 0.352). There were no significant differences regarding surgical complications, post-transplant kidney function (delayed graft function, initial non-function, episodes of acute rejection), or long-term graft survival. Regarding the localization of the arterial anastomosis, non-CIA was an independent prognostic factor for deep vein thrombosis in multivariate analysis (CIA versus non-CIA: OR 11.551; 95% CI, 1.218–109.554; *p* = 0.033). Conclusion: Multiple-donor renal arteries should not be considered a contraindication to deceased KT, as morbidity rates and long-term outcomes seem to be comparable with grafts with single arteries and less complex anastomoses.

## 1. Introduction

Kidney transplantation (KT) is the treatment of choice in patients with end-stage renal disease and it improves patient survival and recipients’ quality of life compared to chronic dialysis treatment [1,2,3].

During kidney graft implantation, vascular anastomosis is one of the most challenging aspects for the transplant surgeon, and post-operative vascular complications such as bleeding or thrombosis can require surgical repair or even nephrectomy [4]. Complex arterial reconstructions are often necessary, as kidney grafts carry two or more arteries in about 30% of cases [5]. However, only a few publications have addressed the issue of multiple renal arteries and complex vascular reconstructions in KT using kidneys from deceased donors (DD). Indeed, most of them investigated KT after living donation (LD) or study groups including both LD and DD KT [6]. This could induce bias, as LD offers better pre-operative planning and pre-selection, as well as better graft outcome and survival rates than DD kidney grafts [1,7].

Therefore, this study aimed to investigate the impact of arterial reconstruction on outcome and graft survival in DD KT.

## 2. Patients and Methods

### 2.1. Data Collection and Study Population

Medical data from all adult patients (≥18 years of age) who underwent initial deceased donor kidney transplantation at the University Hospital of Leipzig between October 1993 and December 2017 were retrospectively analyzed. Our data source comprised a prospectively collected electronic database. Patients undergoing living kidney transplantations, multi-organ transplants, re-transplants, machine preservation of donor kidneys, and transplants with graft anastomosis to the aorta or inferior vena cava were excluded from the study. Living KTs were excluded from the study, as deceased donor organs cannot be compared with living donor organs regarding graft outcome and survival rates. Follow-up data were collected up to March 2020.

Patients were divided into three groups according to the number of arteries and anastomosis: (A) 1 renal artery, 1 arterial anastomosis; (B) >1 renal artery, 1 arterial anastomosis; and (C) >1 renal artery, >1 arterial anastomosis.

Characteristics of the study population included donor and recipient age, gender, body mass index (BMI, weight in kg/height in m^2^), donor cause of death, duration of dialysis, and time on the waiting list. The criteria for expanded criteria donors (ECD) kidneys were donors over 60 years of age or donors between 50 and 59 years of age with at least two of the following three criteria: cerebrovascular death, arterial hypertension, or a donor serum creatinine level >1.5 mg/dL [8]. Peri- and post-transplant data included information on cold (CIT) and warm ischemia time (WIT) of the grafts, duration of operation, and immunosuppressive therapy. CIT is defined as the time that the organ spent in cold preservation solution after removal from the donor. WIT is the time from cross-clamping until cold perfusion, plus the time of implantation (organ out of ice until reperfusion).

### 2.2. Outcome Measures

Outcome data included initial non-function (INF), episodes of acute rejection within 12 months after KT, delayed graft function (DGF), intra- and post-operative complications, date of graft failure, and patient death. INF was defined as dialysis dependence or creatinine clearance ≤20 mL/min at three months post-transplant. Acute rejection episodes were histologically proved. DGF was defined as the requirement of dialysis in the first week following transplantation [9]. Using serum creatinine levels, the estimated glomerular filtration rate (eGFR) was calculated by the Chronic Kidney Disease Epidemiology Collaboration (CKD-EPI) equation (mL/min/1.73 m^2^ of standard body surface area (BSA)) [10]. To reduce errors induced by indexing the glomerular filtration rate for body surface area, GFR was adjusted to individual patient body surface area (eGFR × individual BSA [m^2^]/1.73 m^2^ standard BSA = mL/min) [11,12]. Post-operative complications occurring during the first three months after transplantation were analyzed. Complications included delayed wound healing, wound infection, urine leak, bleeding, and development of hematoma and lymphoceles. New-onset diabetes after transplantation (NODAT) was defined as the need for insulin or oral hypoglycemic drugs. Graft failure was defined as the return to dialysis or re-transplantation (patients who died before graft failure were censored). Post-operative mortality included all deaths occurring within 30 days after surgery.

### 2.3. Organ Procurement and Transplantation

The kidney grafts were procured according to the guidelines provided by Eurotransplant (ET) and transplanted into the iliacal fossa. Kidneys were flushed in situ with cold HTK (histidin-tryptophan-ketoglutarat) solution and explanted. For static cold storage, grafts were immersed in HTK solution at 4 °C and stored in three separate bags, whereas the first bag was filled with preservation solution [13,14]. In general, for two renal arteries, the smaller branch was anastomosed to the side of the larger main renal artery. With two arteries of equal size, both were joined to form an ostium or anastomosed separately. In the case of three renal arteries, depending on the vessel diameter, one or two smaller vessel branches were anastomosed to the side of a main renal artery or reimplanted separately on the iliac vessels when the distance between the vessels was too high. Smaller vessels with high risk of thrombosis were frequently ligated. The preferred approach was reimplantation on a common Carrel patch (small portion of surrounding aorta) to avoid an individual reconstruction and impairment of the diameter of either the donor’s or the recipient’s vessel. In the case of multiple arteries, patches were combined by ex vivo reconstruction on the bench when the distance between the two vessels allowed the patches to be combined without tension. The ureter was implanted into the bladder according to the Lich–Gregoir technique using a double J intraureteral splint [15,16].

### 2.4. Immunosuppression

Immunosuppressive therapy comprised an induction therapy with the interleukin-2 receptor antagonist basiliximab or antithymocyte globulin, followed by triple maintenance immunosuppression comprising calcineurin inhibitors (tacrolimus or cyclosporine), and/or mTOR inhibitors (everolimus or sirolimus), antimetabolites (mycophenolate mofetil), and tapered steroids (prednisolone).

### 2.5. Statistical Analysis

For comparison between the groups, the appropriate statistical significance test, including Student’s *t*-test, the chi-squared test, analysis of variance (ANOVA), the Kruskal–Wallis test, and the Wilcoxon–Mann–Whitney test was used. Univariate and multivariate logistic regression analyses were used to evaluate the association between independent variables and binary outcomes of allograft function, and multivariate Cox proportional hazard analysis was applied to assess independent predictors of kidney graft failure. For multivariate analyses, we used a forward stepwise regression model including only clinically relevant variables and those presenting *p* ≤ 0.05 in univariate analysis. Survival rates were calculated using the Kaplan–Meier analysis, and the log-rank test was applied to test statistical significance. Graft survival was calculated as the time from initial transplant to graft failure (re-start of dialysis), censoring for death with a functioning graft. Patient survival was defined as the time from transplant to patient death, censoring for patients still alive at the time of analysis. If a recipient was alive or lost to follow-up at the time of last contact, then survival time was censored at the time of last contact. SPSS software version 21.0 (SPSS Inc., Chicago, IL, USA) was used for statistical analysis. A *p*-value < 0.05 was considered statistically significant.

### 2.6. Literature Review

A review of the literature on publications reporting results after renal transplantation with multiple renal arteries was performed using the Medline (Pubmed) database. The search algorithm included the following medical subject heading terms: kidney or renal transplant, multiple arteries, anastomosis, artery, and outcome. Articles written in languages other than English, articles without an abstract and/or full text, and those specific to the pediatric population were excluded. A manual search of the reference lists supplemented the electronic results. It yielded 53 potentially relevant original articles, of which 13 met the complete selection criteria (Supplementary Table S1) [17,18,19,20,21,22,23,24,25,26,27,28,29]. Publications were collected up to March 2021.

## 3. Results

### 3.1. Baseline Characteristics

A total of 451 patients receiving primary deceased donor kidney transplantation (KT) were included in the study. The mean follow-up period was 7.6 ± 5.7 years. Among the overall study population, 81.8% of the kidney grafts had one (*N* = 369), 16.4% two (*N* = 74), and 7.8% had three (*N* = 35) renal arteries. In 92.2% (*N* = 416) of the cases, a single arterial anastomosis was performed, in 6.7% (*N* = 30) two were performed, and in 1.1% (*N* = 5) three arterial anastomoses were performed. Donor, recipient, and transplant characteristics according to the different complexity of arterial anastomosis (groups A–C) are summarized in Table 1. The three groups were similar for most of their transplant characteristics. Warm ischemia time significantly increased with the complexity of arterial reconstruction (groups A, B, and C: 40 ± 19 min, 45 ± 19 min, and 50 ± 17 min, respectively; *p* = 0.006). Moreover, a longer operation time was associated with more complex arterial anastomoses, although this was not statistically significant (groups A, B, and C: 175 ± 98 min, 180 ± 35 min, and 210 ± 43 min, respectively; *p* = 0.352).

### 3.2. Outcome

The analysis of post-operative outcome parameters is shown in Table 2. There were neither significant differences regarding post-transplant kidney function parameters nor post-operative complications between the three groups.

In the overall study population, 27 kidneys lost their function in the first three months (initial non-function, INF) (groups A, B, and C: 20, 5, and 2, respectively, *p* = 0.352), whereas permanent lack of graft function from the time of transplantation (primary non-function) was observed in six cases (groups A, B, and C: 4, 1, and 1, respectively; *p* = 0.603).

The most frequent post-operative complications were secondary wound healing (*N* = 97, 21.5%), secondary bleeding and hematoma (*N* = 89, 19.7%), and lymphoceles (*N* = 58, 12.9%). The total number of vascular complications was relatively low (arterial stenosis and/or thrombosis: *N* = 10, 2.2%). No differences in hospital stay (groups A, B, and C: 25 ± 16.2 days, 24 ± 16.5 days, and 22 ± 17.4 days, respectively; *p* = 0.817) were noted.

Eight of the 451 patients (1.8%) died in the immediate post-operative course within the first 30 days after surgery. Overall, post-operative mortality was comparable between the groups (groups A, B, and C: 5, 2, and 1, respectively; *p* = 0.322). The causes of death included multiple organ failure (*N* = 2), septic shock (*N* = 1), intracerebral hemorrhage (*N* = 1), and fatal heart attack (*N* = 1) in group A; decompensated heart failure (*N* = 1) and acute bleeding with cardiovascular arrest (*N* = 1) in group B; and decompensated heart failure (*N* = 1) in group C.

Univariate regression analyses of post-operative outcomes associated with the complexity of arterial anastomosis (groups A–C) and anastomosis localization are displayed in Table 3. Patients in group B were more likely to have renal artery thrombosis (OR 5.422; 95% CI: 0.882–33.342; *p* = 0.068), whereas no case of thrombosis was reported in group C. There was no significant difference between the two groups regarding post-transplant kidney function parameters (INF, DGF, acute rejection).

Furthermore, in the univariate regression model, outcome parameters significantly associated with the localization of arterial anastomoses (CIA versus non-CIA) were thrombosis of the renal artery (OR 4.730; 95% CI 1.041–21.500; *p* = 0.044) and deep-vein thrombosis (OR 7.116; 95% CI 1.284–39.444; *p* = 0.025). Univariate risk analysis showed no correlation between cause of renal failure, warm ischemia time, the use of arterial patches, and post-operative outcome parameters (Supplementary Table S2).

In the multivariate regression analysis, complexity of arterial reconstruction (groups A–C) failed to be an independent predictor of post-operative complications and graft function. However, multivariate analysis showed that the localization of arterial anastomoses is an independent prognostic factor for deep-vein thrombosis (OR 11.551; 95% CI, 1.218–109.554; *p* = 0.033) (Table 4).

### 3.3. Graft and Patient Survival

The one-, three-, five-, and 10-year kidney graft survival rates were 96%, 89%, 85%, and 72% in group A, 91%, 91%, 85%, and 78% in group B, and 88%, 85%, 81%, and 81% in group C (*p* = 0.733), respectively. Mean kidney graft survival decreased with the increasing complexity of arterial anastomoses, although these trends were not statistically significant among the groups (groups A, B, and C: 191 ± 7.1 months, 182 ± 14.6 months, and 147 ± 11.3 months, respectively; *p* = 0.733). Overall long-term patient survival at one, three, five, and 10 years was 95%, 90%, 87%, and 74% in group A, 96%, 91%, 91%, and 83% in group B, and 94%, 91%, 88%, and 80% in group C (*p* = 0.633), respectively.

## 4. Discussion

Multiple renal arteries are the Achilles heel of KT and lead to complications, especially during graft implantation and the early post-transplant course. In our series, 18.2% of the kidney grafts had multiple renal arteries, and in about 7.7% of the cases more than one arterial anastomosis was created. These results are comparable with reports of autopsies and previous studies in KT [5,17,18,19,20,21,22,23,24,25,26,27,28,29].

### 4.1. Vascular Complications

Most of the previously published reports showed higher rates of vascular complications in kidney grafts with multiple renal arteries (MRA) compared to single anastomosis [17,18,19,20,21,22,23,24,25,26,27,28,29]. Consecutive inadequate perfusion can lead to transplant infarction, ureteral necrosis, and a higher rate of graft loss. Renal artery stenosis occurred among 0.1% to 18.8% of KT with MRA and became primarily symptomatic through hypertension and functional impairment of the graft six months to three years after KT [17,19,21,24]. In a study published by Benedetti et al. in 1998 investigating the outcome of approximately 1000 KT, MRA was an independent risk factor for arterial stenosis in multivariate analysis (*p* = 0.04). Interestingly, arterial stenosis only occurred in cases with MRA on common aortic patches, not on reconstructed MRA patches [17]. In our series, the most common vascular complications were arterial thromboses, although no significant differences or independent effects in the adjusted analysis were observed among groups A–C. However, the localization of renal artery anastomosis seems to have a significant impact on the thrombosis rate. Usually, end-to-side anastomosis between the renal artery and the common iliac artery (CIA) is performed. Less frequently, the renal artery is anastomosed with the external iliac artery, or an end-to-end reconstruction is created with the internal iliac artery. Moreover, as described in the literature, end-to-end anastomosis to the internal iliac could increase the risk of erectile dysfunction in men [30]. In our series, anastomoses to the external or internal iliac artery were associated with a higher risk of arterial thrombosis and deep-vein thrombosis. Therefore, anastomoses to the CIA should be preferred whenever possible.

### 4.2. Delayed Graft Function

In the reviewed studies investigating KT after deceased donation, two reports showed delayed graft function (DGF) data with a slight insignificant increase in DGF in the MA groups [22,26]. DGF is thought to be a result of immunologically and ischemic-induced graft injury [7,9]. However, although reconstruction of multiple arteries led to a significantly prolonged time of warm ischemia and increased time of surgery in our cohort, rates of delayed graft function (DGF) were comparable among groups A–C (complexity of arterial reconstruction). Other donor characteristics like the type of donation could trigger DGF. In general, the percentage of DGF after deceased donor KT is inherently higher compared to living donor KT. The prevalence of DGF ranged from 4% to 10% in patients after living KT, and between 2% and 50% in kidneys from brain-dead donors [7,31]. Interestingly, a meta-analysis by Zorgdrager et al. showed significantly more DGF in MA grafts than in grafts with single renal arteries but failed to be significant when studies with deceased donor grafts were excluded [6]. However, the available data are incomplete, and studies do not allow a linkage to be made between donor type and DGF, which probably caused significant heterogeneity in the analysis.

### 4.3. Graft Survival

Long-term outcomes and especially graft survival rates seem to be comparable between kidney grafts with single and multiple arteries. However, most published studies investigated KT after living donation (LD) or mixed cohorts comprising both LD and DD KTs [6,17,18,19,20,21,22,23,24,25,26,27,28,29]. This critical difference could have induced bias, as LD kidney grafts cannot be compared to DD kidney grafts regarding graft outcome and survival rates [1,7,32]. Moreover, compared to DD, LD offers better pre-operative planning and pre-selection, whereby the total ischemia time is usually shorter in LD KT.

### 4.4. Limiting Factors

There are some limiting factors of this study. First, one limitation of our study is the relatively low number of patients with multiple arteries and complex arterial anastomoses and its monocentric, retrospective non-randomized design. Second, the long investigation period makes further controlled and prospective studies necessary.

## 5. Conclusions

In conclusion, based on our data, complex arterial reconstructions of multiple renal arteries are not a limiting factor in kidney transplants regarding early post-operative outcomes and long-term graft survival. However, the localization of the renal artery anastomosis was associated with deep-vein thrombosis, and anastomoses to the internal or external iliac recipient artery should be avoided whenever possible.

## Figures and Tables

**Table 1:** Donor, recipient, and transplant characteristics.

Variables			Group			*p*-Value
			A (*N* = 369)	B (*N* = 47)	C (*N* = 35)	
Donor						
	Age, years		53 ± 17.6	49 ± 18.4	57 ± 14.1	0.429
	Gender, male/female (%)		201 (54.5)/159 (43.1)	30 (63.8)/17 (36.2)	18 (51.4)/17 (48.6)	0.514
	BMI, kg/m^2^		24.8 ± 3.9	24.5 ± 3.2	24.8 ± 4.7	0.672
	Cause of death (%)					
		CAV	200 (54.2)	22 (46.8)	16 (45.7)	0.152
		Non-CAV, anoxia/ischemia/polytrauma/others/unknown	35 (9.5)/34 (9.2)/50 (13.6)/15 (4.1)/1 (0.3)	3 (6.4)/7 (14.9)/6 (12.8)/2 (4.3)/1 (2.1)	1 (2.9)/7 (20.0)/3 (8.6)/4 (11.4)/0	
	Comorbidity (%)					
		Diabetes mellitus	32 (8.7)	5 (10.6)	2 (5.7)	0.734
		Hypertension	136 (36.9)	21 (44.7)	12 (34.3)	0.534
	ECD (%)		157 (42.5)	19 (40.4)	17 (48.6)	0.743
Recipient						
	Age, years		55.9 ± 13.2	53.2 ± 14.4	53.6 ± 11.7	0.652
	Gender, male/female (%)		213 (57.7)/138 (37.4)	31 (66.0)/16 (34.0)	17 (48.6)/18 (51.4)	0.219
	BMI, kg/m^2^		25.0 ± 4.2	24.8 ± 3.9	25.2 ± 3.1	0.576
	Cause of ESRD (%)					
		Glomerulonephritis	126 (34.1)	21 (44.7)	11 (31.4)	0.013
		Non-glomerulonephritis, diabetes mellitus/cystic kidney disease/interstitial nephritis/others	24 (6.5)/58 (15.7)/43 (11.7)/118 (32.0)	3 (6.4)/14 (29.8)/1 (2.1)/8 (17.0)	0/12 (34.3)/0/12 (34.3)	
	Comorbidity (%)					
		Diabetes mellitus	65 (17.6)	4 (8.5)	4 (11.4)	0.201
		Hypertension	342 (92.7)	41 (87.2)	32 (91.4)	0.427
		Coronary disease	53 (14.4)	11 (23.4)	8 (22.9)	0.143
		PVD	28 (7.6)	3 (6.4)	3 (8.6)	0.930
Transplant						
	Transplant era (%)					
		1993–2001/2002–2009/2010–2017	126 (34.1)/120 (32.5)/123 (33.3)	17 (36.2)/19 (40.4)/11 (23.4)	7 (20.0)/15 (42.9)/13 (37.1)	0.271
	CIT, hours		12.5 ± 6.2	11.4 ± 4.5	11.3 ± 5.5	0.421
	Number renal arteries, 1/2/3 (%)		369 (100)/0/0	0/44 (93.6)/3 (6.4)	0/30 (85.7)/5 (14.3)	<0.001
	Arterial anastomosis (%)					
		Number, 1/2/3	369 (100)/0/0	47 (100)/0/0	0/30 (85.7)/5 (14.3)	<0.001
		Location, IIA/EIA/CIA	8 (2.2)/67 (18.2)/283 (76.7)	2 (4.3)/8 (17.0)/36 (76.6)	0/14 (40.0)/21 (60.0)	0.033
		Patch	215 (58.3)	39 (83.0)	22 (62.9)	0.005
	WIT, minutes		40 ± 19	45 ± 19	50 ± 17	0.006
	Ureterocystoneostomy (%)		343 (93.0)	45 (95.7)	34 (97.1)	0.876
	Intra-operative complications (%)					
		Bleeding	16 (4.3)	1 (2.1)	3 (8.6)	0.366
		Thrombosis renal artery	10 (2.7)	1 (2.1)	1 (2.9)	0.970
		Thrombosis renal vein	6 (1.6)	0	0	0.509
	Duration of surgery, minutes		175 ± 98	180 ± 35	210 ± 43	0.352

Data are shown as median ± SD. BMI, body mass index; CIA, common iliac artery; CIT, cold ischemia time; CVA, cerebrovascular event; ECD, expanded criteria donors; ESRD, end-stage renal disease; EIA, external iliac artery; IIA, internal iliac artery; PVD, peripheral vascular disease; WIT, warm ischemia time.

**Table 2:** Post-operative outcome parameters and immunosuppression after deceased donor kidney transplantation.

Variables				Group			*p*-Value
				A (*N* = 369)	B (*N* = 47)	C (*N* = 35)	
Kidney function							
	INF (%)			20 (5.4)	5 (10.6)	2 (5.7)	0.351
	DGF (%)			109 (29.5)	12 (25.5)	12 (34.3)	0.726
	Acute rejection (%)			101 (27.4)	11 (23.4)	9 (25.7)	0.815
	Laboratory tests						
		GFR (mL/min), POD7		30.5 ± 37.3	40.0 ± 36.6	22.0 ± 33.0	0.919
		GFR (mL/min), POM1		74.4 ± 36.5	86.2 ± 39.6	76.9 ± 41.9	0.237
Surgical outcome							
	RBC substitution (%)			106 (28.7)	18 (38.3)	10 (28.6)	0.396
	Time in ICU, days			5 ± 5.7	5 ± 3.3	5 ± 2.5	0.471
	Hospitalization, days			25 ± 16.2	24 ± 16.5	22 ± 17.4	0.817
	Post-operative complications (%)						
		Deep-vein thrombosis		5 (1.4)	0	1 (2.9)	0.533
		Stenosis					
			Renal artery	2 (0.5)	0	0	0.800
			Renal vein	1 (0.3)	0	0	0.895
		Thrombosis					
			Renal artery	3 (0.8)	2 (4.3)	0	0.085
			Renal vein	7 (1.9)	1 (2.1)	1 (2.9)	0.925
		Bleeding/hematoma		68 (18.4)	12 (25.5)	9 (25.7)	0.335
		Secondary wound healing		80 (21.7)	12 (25.5)	5 (14.3)	0.463
		Burst abdomen		7 (1.9)	1 (2.1)	1 (2.9)	0.925
		Urine leakage		13 (3.5)	1 (2.1)	0	0.473
		Lymphocele		47 (12.7)	4 (8.5)	7 (20.0)	0.303
Metabolic							
	NODAT (%)			21 (5.7)	0	4 (11.4)	0.079
Immunosuppression							
	Induction therapy (%)			129 (35.0)	15 (31.9)	15 (42.9)	0.676
	Maintenance therapy (%)						
	CNI, Tac/CsA			209 (56.6)/148 (40.1)	28 (59.6)/17 (36.2)	25 (71.4)/9 (25.7)	0.184
	mTOR inhibitor, Ever/Siro			2 (0.5)/9 (2.4)	0/0	0/1 (2.9)	0.833
	CNI + mTOR inhibitor			11 (3.0)	0	1 (2.9)	0.491
	AM drug, MMF (%)			326 (88.3)	43 (91.5)	34 (97.1)	0.223
	Steroids, prednisolone			353 (95.7)	43 (91.5)	33 (94.3)	0.361
Post-operative mortality (%)				5 (1.4)	2 (4.3)	1 (2.9)	0.322

Data are shown as median ± SD. AM, antimetabolite; CNI, calcineurin inhibitor; CsA, ciclosporin A; DGF, delayed graft function; Ever, everolimus; GFR, glomerular filtration rate; ICU, intensive care unit; INF, initial non-function; MMF, mycophenolate mofetil; mTOR, mechanistic target of rapamycin; NODAT, new-onset diabetes mellitus after transplantation; POD, post-operative day; POM, post-operative month; RBC, red blood cells; Siro, sirolimus; Tac, tacrolimus.

**Table 3:** Univariate analysis of kidney transplant outcome according to the complexity of arterial reconstruction and localization of arterial anastomosis.

Variables		Group							Location of Arterial Anastomosis			
		A	B			C			CIA	Non-CIA		
			Univariate Analysis			Univariate Analysis				Univariate Analysis		
			OR	95% CI	*p*	OR	95% CI	*p*		OR	95% CI	*p*
Complications												
	RBC substitution	1.00	1.540	0.820–2.891	0.400	0.992	0.461–2.138	0.985	1.00	0.960	0.585–1.577	0.872
	Thrombosis renal artery	1.00	5.422	0.882–33.324	0.068	N/A	N/A	N/A	1.00	4.730	1.041–21.500	0.044
	Thrombosis renal vein	1.00	1.124	0.135–9.344	0.914	1.521	0.182–12.730	0.699	1.00	0.375	0.047–2.999	0.355
	Secondary bleeding	1.00	1.518	0.749–3.076	0.247	1.532	0.687–3.418	0.297	1.00	0.950	0.539–1.674	0.859
	Deep-vein thrombosis	1.00	N/A	N/A	N/A	2.141	0.243–18.859	0.493	1.00	7.116	1.284–39.444	0.025
Kidney function												
	INF	1.00	2.104	0.749–5.905	0.158	1.045	0.234–4.670	0.954	1.00	1.598	0.673–3.796	0.288
	DGF	1.00	0.829	0.414–1.661	0.597	1.225	0.589–2.551	0.587	1.00	0.730	0.436–1.222	0.231
	Acute rejection	1.00	0.806	0.394–1.648	0.554	0.888	0.402–1.960	0.768	1.00	0.613	0.351–1.068	0.084
Graft failure		1.00	0.751	0.363–1.553	0.440	0.918	0.399–2.110	0.840	1.00	0.856	0.483–1.516	0.594

95% CI, 95% confidence interval; CIA, common iliac artery; DGF, delayed graft function; EIA, external iliac artery; IIA, internal iliac artery; INF, initial non-function; N/A, not available; OR, odds ratio; RBC, red blood cells.

**Table 4:** Multivariate analysis of kidney transplant outcome parameters.

Variables		Thrombosis Renal Artery			Deep-Vein Thrombosis		
		OR	95% CI	*p*-Value	OR	95% CI	*p*-Value
Arterial patch		0.339	0.015–7.816	0.499	0.616	0.095–3.988	0.611
Location of arterial anastomosis (CIA vs. Non-CIA)		2.864	0.165–49.692	0.470	11.551	1.218–109.554	0.033
Group							
	A	ref			ref		
	B	13.595	0.577–320.067	0.105	N/A	N/A	N/A
	C	N/A	N/A	N/A	1.061	0.101–11.096	0.611
WIT		0.735	0.040–13.446	0.835	1.332	0.203–8.747	0.765

95% CI, 95% confidence interval; OR, odds ratio; WIT, warm ischemia time.

## Data Availability

Our database contains highly sensible data that may provide insight into clinical and personnel information about our patients and lead to identification of these patients. Therefore, according to organizational restrictions and regulations these data cannot be made publicly available. However, the datasets used and/or analyzed during the current study are available from the corresponding author on reasonable request.

## Supplementary Materials

The following are available online at https://www.mdpi.com/article/10.3390/jcm10194395/s1, Table S1: Previous publications (1995–2021) investigating influence of multiple renal arteries in study groups including deceased donor kidney transplantation, Table S2: Univariate analysis of kidney transplant outcome according to the cause of renal failure, warm ischemia time and the use of arterial patches for anastomoses.
