# Supplementary material for: Influence of Multiple Donor Renal Arteries on the Outcome and Graft Survival in Deceased Donor Kidney Transplantation
Source: J Clin Med. 2021 Sep 26;10(19):4395. doi: 10.3390/jcm10194395 (PMC8509629; doi:10.3390/jcm10194395)
Supplement: Supplementary file 1 [file jcm-10-04395-s001.zip › jcm-1370771-supplementary.pdf]

Table S1. Previous publications (1995-2021) investigating influence of multiple renal arteries in study groups including deceased donor kidney transplantation.

Author	Period	Groups	Patients (%)	DD (%)	CIT, hours ± SD	WIT, minutes ± SD	Vascular complication, artery (%)		Lymphocele (%)	Urine leakage (%)	DGF (%)	Serum creatinine, mg/dL		Creatinine clearance, mL/ min		Graft survival rates (%)		
							Stenosis	Thrombosis				1 month	1 year	1 month	1 year	1 year	5 year	10 year
Benedetti et al. [17]	1985 -1993	SA	835 (84)	47			13 (1.4)#	4 (0.5)		8 (1)			1.66			88	72	N/A
		MA, 1 anast.	112 (11)	91	N/A	N/A	6 (5.4)	-	N/A	1 (0.9)	N/A	N/A	1.7	N/A	N/A	94	73	N/A
		MA, >1 anast.	51 (5)	18			1 (2)	-		1 (2)			1.76			83	77	N/A
Han et al. [18]	1990 -1996	SA	435 (87)	N/A	LD/ DD: 0.5*/ 10.4 (TIT)		-	-	N/A	1 (0.2)	N/A	N/A	N/A	N/A	N/A	96	N/A	N/A
		MA	65 (13)		LD/ DD: 1.2/ 10.6		-	-		3 (4.6)						97		
Emiroğlu et al. [19]	1975 -1999	SA anast.	861 (92)	17	single/ double/ triple artery anastomosis: 1.0 ± 0.2 / 1.4 ± 0.3/ 1.5 ± 0	N/A	N/A	N/A	N/A	N/A	N/A	N/A	N/A	N/A	N/A	95	79	N/A
		MA anast.	74 (8)	20			3 (4.1)	-	1 (1.4)	-						95	72	
Aydin et al. [20]	N/A	SA	196 (87)	N/A	N/A	N/A	N/A	N/A	N/A	N/A	N/A	N/A	N/A	N/A	N/A	95	73	N/A
		MA	29 (13)	28			-	-	-	-			1.98 ± 0.72			93	78	
Başaran et al. [21]	1975 -2003	SA	1016 (93)	17	1.5 ± 0.2	N/A	N/A	N/A	N/A	N/A	N/A	N/A	1.0 ± 0.7	N/A	N/A	95	79	N/A
		MA	79 (7)	23	1.4 ± 0.2		3 (3.8)	2 (2.5)	2 (2.5)				1.1 ± 0.5			95	73	
Mazzucchi et al. [22]	1995 -1999	SA	292 (82)	64	N/A	N/A	6 (2.1)	1 (0.3)	9 (3.1)**	8 (2.7)	85 (29.1)	1.81	N/A	N/A	N/A	N/A	N/A	N/A
		MA	64 (18)	63			-	-	8 (12.5)	3 (4.7)	23 (35.1)	2.46						
Gawish et al. [23]	1993 -2005	SA	611 (95)	26	N/A	N/A	13 (2.1)	16 (2.6)	N/A	N/A	N/A	1.31 ± 0.6	1.38 ± 1.0	N/A	N/A	94	88	84
		MA	35 (5)	51			1 (2.9)	1 (2.9)				1.39	1.58			94	89	83
Hwang et al. [24]	1990 -2008	SA	890 (75)		1.2 ± 1.5***(TIT)		16 (1.3)	1 (0.1)	17 (1.4)	10 (0.8)		1.29 ± 0.81	1.43 ± 0.98			92	83	73
		MA, 1 anast.	26 (2)		2.2 ± 2.6		-	-	2 (0.2)	-		1.19 ± 0.31	1.42 ± 0.70			83	71	62
		MA, >1 anast.	236 (20)	16 total	1.1 ± 1.2		1 (0.1)	-	8 (0.7)	5 (0.4)	N/A	1.38 ± 1.21	1.52 ± 1.39	N/A	N/A	91	81	73
		MA, ligation polar artery	34 (3)		1.5 ± 2.1		-	-	1 (0.1)	1 (0.1)		1.52 ± 1.04	1.39 ± 0.68			88	85	80
Vasquez et al. [25]	2006 -2008	SA	70 (81)	20	N/A	112 ± 84*	3 (4.5)	N/A	N/A	N/A	N/A	1.2 ± 0.4	1.2 ± 0.4	N/A	N/A	N/A	N/A	N/A
		MA	16 (19)	38		216 ± 168	3 (18.8)					1.5 ± 0.5	1.5 ± 0.6					
Laouad et al. [26]	1996 -2000	SA	189 (73)	100	20.6 ± 5.9	54.3 ± 17.9	8 (4.2)	4 (2.1)	N/A	4 (2.1)	37 (19.8)	N/A	N/A	N/A	60.0 ± 20.3	N/A	81	41
		MA	70 (27)	100	20.5 ± 6.6	51.8 ± 20.4	4 (5.7)	2 (2.9)		1 (1.4)	17 (24.6)				64.1 ± 19.7		81	29
Sezer et al. [27]	2000 -2005	SA	214 (86)	51	N/A	N/A	N/A	-	14 (6.5)	10 (4.6)	N/A	N/A	N/A	N/A	71 ± 24	N/A	90	N/A
		MA	35 (14)	34				1 (2.8)	2 (5.7)	2 (5.7)					67 ± 26		91	
Bozkurt et al. [28]	2006 -2012	MA	196 (100)	16		N/A	-	-	3 (1.5)	1 (0.5)	N/A	N/A	N/A	N/A	N/A	N/A	N/A	N/A
Sevmis et al. [29]	2016 -2017	SA	165 (85)	11 total	N/A	N/A	N/A§	-	NS	NS	7 (4.2)	N/A	N/A	72.0 ± 23.1	71.3 ± 25.0	96	N/A	N/A
		MA	30 (15)				-	-			-			65.8 ± 19.1	68.1 ± 20.8	94		
Present study	1993 -2017	SA	369 (82)	100	12.5 ± 6.2	40 ± 19**	2 (0.5)	3 (0.8)	47 (12.7)	13 (3.5)	109 (29.5)			74.4 ± 36.5		96	85	72
		MA, 1 anast.	47 (10)	100	11.4 ± 4.5	45 ± 19	-	2 (4.2)	4 (8.5)	1 (2.1)	12 (25.5)	N/A	N/A	86.2 ± 39.6	N/A	91	85	78
		MA, >1 anast.	35 (8)	100	11.3 ± 5.5	50 ± 17	-	-	7 (20)	-	12 (34.3)			76.9 ± 41.9		88	81	81

Data are shown as median ± standard deviation (SD). DD, deceased donor; DGF, delayed graft function; MA, multiple arteries, N/A, not available; NS, not significant; SA, single artery; TIT, total ischemic time; s#, SA versus MA, 1 anastomosis: P = 0.0196, MA, 1 anastomosis versus MA, >1 anastomosis: P = 0.5966; §, unspecified vascular injury, 1 (0.5); *, P < 0.05; **, P < 0.01.

Table S2. Univariate analysis of kidney transplant outcome according to the cause of renal failure, warm ischemia time and the use of arterial patches for anastomoses.

Variables	Cause of ESRD (Non-glomerulonephritis vs glomerulonephritis)			WIT			Arterial patch		
	OR	95% CI	P-value	OR	95% CI	P-value	OR	95% CI	P-value
Complications									
RBC substitution	1.050	0.689–1.602	0.820	0.827	0.473–1.445	0.504	0.956	0.632–1.446	0.832
Thrombosis renal artery	2.816	0.466–17.033	0.260	0.952	0.059–15.389	0.972	0.418	0.069–2.530	0.343
Thrombosis renal vein	0.457	0.096–2.177	0.325	0.951	0.132–6.864	0.960	0.627	0.179–2.199	0.466
Secondary bleeding	0.930	0.570–1.517	0.770	1.776	0.952–3.313	0.071	0.897	0.641–1.663	0.897
Deep vein thrombosis	1.871	0.373–9.380	0.446	1.439	0.236–8.764	0.693	0.312	0.057–1.722	0.181
Kidney function									
INF	0.771	0.330–1.804	0.549	1.966	0.576–6.709	0.280	0.568	0.260–1.239	0.155
DGF	1.023	0.669–1.564	0.917	1.240	0.709–2.169	0.451	0.897	0.593–1.358	0.609
Acute rejection	0.801	0.514–1.250	0.329	1.016	0.577–1.788	0.957	1.046	0.682–1.606	0.836

95% CI, 95% confidence interval; DGF, delayed graft function; INF, initial non-function; OR, odds ratio; RBC, red blood cells; WIT, warm ischemia time.
